# Impact of *IL8 *and *IL8-Receptor alpha *polymorphisms on the genetics of bronchial asthma and severe RSV infections

**DOI:** 10.1186/1476-7961-4-2

**Published:** 2006-02-17

**Authors:** Beena Puthothu, Marcus Krueger, Jessica Heinze, Johannes Forster, Andrea Heinzmann

**Affiliations:** 1 University Children's Hospital, University of Freiburg, Mathildenstrasse 1, D-79106 Freiburg, Germany; 2 St. Josefhospital, Sautier Str. 1, D-79104 Freiburg, Germany

## Abstract

**Background:**

Interleukin 8 (IL8) belongs to the family of chemokines. It mediates the activation and migration of neutrophils from peripheral blood into tissue and hereby plays a pivotal role in the initiation of inflammation. Thus it is important in inflammatory lung diseases like bronchial asthma or severe infections by Respiratory Syncytial Virus (RSV). IL8 acts through binding to the IL8-Receptor alpha (IL8RA). For both genes association with asthma has been described. In addition, *IL8 *has been found in association with RSV bronchiolitis. The aim of our study was to test both genes for association with asthma and severe RSV infections. In addition we were interested in whether a common genetic background of both diseases exists in regards to these genes.

**Methods:**

We genotyped the two *IL8 *promotor polymorphisms -251A/T and -781C/T and the three amino acid variants M31R, S276T and R335C in *IL8RA *on 322 children with asthma, 131 infants with severe RSV associated diseases and 270 controls. Statistical analyses made use of the Armitage's trend test for single polymorphisms and FAMHAP for calculations of haplotypes.

**Results:**

We found association of the *IL8 *polymorphism -781C/T as well as *IL8 *haplotypes with asthma (p = 0.011 and p = 0.036, respectively). In addition, direct comparison of the asthmatic population with the RSV population revealed significant differences, both for -781C/T alone (p = 0.034) and *IL8 *haplotypes (p = 0.005). The amino acid variants in IL8RA were evenly distributed in between all three populations.

**Conclusion:**

We conclude from our data that *IL8 *might play a role in the genetic predisposition to asthma and that these effects are different or even opposite to the effects on severe RSV diseases. Furthermore, IL8RA is unlikely to play a major role in the genetics of either disease.

## Background

Interleukin 8 (IL8) is a member of the chemokine family and is produced by a wide range of cell types like monocytes, macrophages, fibroblasts and ceratinocytes. It primarily mediates the activation and migration of neutrophils from peripheral blood into tissue and is involved in the initiation and amplification of inflammatory processes, which occur in the human immune system in response to a wide variety of pathogens [1]. Thus IL8 play an important role in inflammatory lung diseases like bronchial asthma or severe infections caused by respiratory syncytial virus (RSV).

RSV, a single-stranded RNA virus, is involved in at least 70% of cases of infectious infantile bronchiolitis and has been repetitively linked to asthma. It has been hypothesized that severe RSV infection in infancy might lead to the development of recurrent wheezing and/or bronchial asthma [2,3], and consequently a common genetic background of both diseases has been discussed [4]. According to the current evidence, genetic and environmental factors determine the type of immune response to RSV infection. Furthermore, this response may affect the development of control mechanisms in the regulation of airway diseases, mainly bronchial asthma.

Increased concentrations of IL8 have been described in the bronchoalveolar fluid and sputum of asthmatic patients [5]. In addition, repeated administration of IL8 into the airways induces bronchial hyperreactivity in guinea pigs [6,7]. Genetic association of IL8 has been described with asthma [8] and RSV bronchiolitis [9,10].

IL8 binds with high affinity to two different receptors: IL8 receptor alpha (IL8RA, CXCR1) and beta (IL8RB, CXCR2). These closely related proteins are members of the superfamily of receptors, which couple to guanine nucleotide binding proteins. *IL8RA *is localized on chromosome 2q35 [11], where linkage to total serum IgE levels in asthmatics has been described [12]. In addition, association of *IL8RA *polymorphisms has recently been described with asthma and chronic obstructive pulmonary disease [13].

We were interested in the relationship between severe RSV infection and/or bronchial asthma and *IL8 and IL8RA *polymorphisms in the German population. We chose to study two *IL8 *promoter polymorphisms (-251A/T and -781C/T) and three amino acid variants in *IL8RA *(M31R, S276T and R335C), which are located in the N-terminus of the protein, in the third extracellular loop and in the C-terminus of the protein respectively. The selection of the *IL8 *polymorphisms was based on previous studies in our asthmatic and control populations, in which these polymorphisms were shown to be most informative [8].

## Materials and methods

### Subjects

#### Asthmatic population

322 children with bronchial asthma (aged 5 to 18 years) were recruited from the South-Western part of Germany between July 2000 and January 2005. All probands were characterized at the University Children's Hospital, Freiburg, Germany using a standardized clinical protocol. An extended medical history was recorded including the occurrence and duration of wheezing symptoms; previous and acute medications; severity of previous asthma attacks; previous allergic rhinitis or conjunctivitis; atopic dermatitis and any family history of allergic diseases.

The diagnosis was based on a clear-cut history of asthmatic symptoms; the use of anti-asthmatic medication and the presence of bronchial hyper-reactivity. The anti-asthmatic drugs included typical betamimetics like salbutamol and standard corticosteroids used in asthma treatment like budesonide. Bronchial hyper-reactivity was defined as a fall in forced respiratory volume in one second (FEV1) by at least 15% in histamine testing or exercise provocation, using standard protocols [14]. The exact recruitment procedure has been described in detail previously [15].

#### Population of children with severe RSV infection

This population was recruited at the University Children's Hospital, Freiburg, Germany and also in the Community Children's Hospital, St. Josef's Hospital, Freiburg. Infants and children were eligible when hospitalized due to RSV infection between September 1998 and March 2005 at an age of less than 2 years. RSV infection was detected by antigen test and/or RSV-specific PCR [16]. According to the case definition, children had symptoms of bronchiolitis, for example wheezing and tachypnoe and needed either supplementary oxygen and/or gavage feeding and/or intravenous fluids. Children with congenital heart defects, immune deficiency or chromosomal aberrations were excluded. DNA samples were collected either by blood taking or buccal smears with sterile cotton sticks. In total 131 children were included in this study.

### Control population

270 randomly chosen probands, aged 19 to 40 years, served as controls. They originated from the same area in the South-Western part of Germany. No medical history was taken and no medical testing was performed on controls.

### Genotyping

DNA was extracted from peripheral blood leucocytes or buccal smears following standard protocols and column purified (DNA midikit, Qiagen, Germany). The two *IL8 *polymorphisms (-251A/T and -781C/T) were typed as described previously [8].

The three *IL8R *polymorphisms were typed by means of restriction fragment length polymorphism (RFLP):

Met31Arg (rs16858811) was typed using the primer pair 5'-TGAAGATTACAGGCCCTGTA-3' and 5'-AAATCCAGCCATTCACCTTG-3'. Following PCR, the product was digested with two units of BglI (Fermentas) at 37° over night and the fragments were resolved on a 4% agarose gel.

Ser276Thr (rs16858809) was typed using the primer pair 5'-TCACCCTGCGTACACTGTTT-3' and 5'-GCCAAGAACTCCTTGCTGAC-3. Following PCR, the product was digested with one unit of Alw261 (Fermentas) at 37°C over night and the fragments were resolved on a 4% agarose gel.

Arg335Cys (rs16858808) was typed using the primer pair 5'-AGGAGTTCTTGGCACGTGAT -3' and 5'-AATGATGGTGCTTCGTTTCC-3'. Following PCR, the product was digested with one unit of DpnII (NEB) at 37°C over night and the fragments were resolved on 4% agarose gel.

### Sequencing

For each polymorphism, three controls (homozygous wild type, heterozygous and homozygous mutation) were sequenced by the dideoxy chain termination method [17], using the Big Dye Terminator cycle sequencing kit on an ABI 310 sequencer (Applied Biosystems). All the following studies included these reference individuals.

### Statistical analysis

Association analysis was performed for each polymorphism using Armitage's Trend Test. This test takes into account the individuals' genotypes rather than just the alleles, following the guidelines given by Sasieni [18], as implemented in the program Finetti (Thomas F. Wienker, unpublished data;  and ). Hardy Weinberg equilibrium (HWE) was calculated for each polymorphism in all three populations using also the program Finetti. In addition to analyses based on single polymorphisms, we performed haplotype frequency estimations using the program FAMHAP [19]. The extent of linkage disequilibrium between polymorphisms has been calculated by Arlequin.

### Approval

The collection of blood and the experimental procedures were approved by the Ethical Committee of the University of Freiburg. A statement of informed consent was signed by all participants; or in the case of children, signed by their parents.

## Results

### Genotyping

Five polymorphisms, two *IL8 *and three *IL8RA *polymorphisms, were typed on 131 children with severe RSV infection, 270 controls and 322 asthmatic children. The allelic frequencies of the polymorphisms in the three populations and the Hardy Weinberg equilibrium are given in table 1. All polymorphisms were in Hardy Weinberg equilibrium in all populations.

**Table 1:** Frequency and HWE of the polymorphisms within the three populations.

**Polymorphism**	**Asthma Frequency**	**HWE**	**RSV Frequency**	**HWE**	**Controls Frequency**	**HWE**
*IL8 *rs4073 (-251A/T)	0.441	0.469	0.470	0.241	0.493	0.223
*IL8 *rs2227306 (-781C/T)	0.618	0.708	0.540	0.914	0.543	0.242
*IL8RA *rs16858811 (M31R)	0.969	1.000	0.974	1.000	0.954	1.000
*IL8RA *rs16858809 (S276T)	0.933	1.000	0.948	1.000	0.944	1.000
*IL8RA *rs16858808 (R335C)	0.967	1.000	0.974	1.000	0.954	0.441

Results of genotyping of the *IL8 *and *IL8RA *variants on the three populations. The frequency is given for the wildtype allele. Also given is the p-value for the Hardy Weinberg Equilibrium (HWE) as calculated by Finetti.

### Association studies

The genotype distribution in the populations and the p-values for association, obtained by Armitage's Trend Test, are listed in table 2. An association was observed between asthma and the promoter polymorphism -781C/T in *IL8 *(p = 0.011). When comparing children with asthma to children with severe RSV infection, a significant different allelic distribution was observed for the same polymorphism (p = 0.0342). The other evaluated polymorphisms showed no association with bronchial asthma or severe RSV infection.

**Table 2:** Genotype distribution and p-values for association

**Polymorphism**	**Genotype distribution**			**p-values for association**		
	Asthma	RSV	Controls	Asthma- Controls	RSV- Controls	Asthma- RSV
*IL8 *rs4073 (-251A/T)	64; 148; 101	26; 73; 34	70; 124; 74	0.087	0.550	0.425
*IL8 *rs2227306 (-781C/T)	123; 147; 48	37; 62; 27	84; 124; 61	0.011	0.937	0.034
*IL8RA *rs16858811 (M31R)	299; 20; 0	128; 7; 0	245; 25; 0	0.174	0.152	0.655
*IL8RA *rs16858809 (S276T)	239; 30; 0	121; 14; 0	121; 14;0	0.409	0.812	0.376
*IL8RA *rs16858808 (R335C)	298; 21; 0	127; 7; 0	246; 23; 1	0.239	0.171	0.584

The genotype distribution within the three different populations is given in the following order: homozygous wildtype, heterozygous and homozygous mutation. Also listed are the p-values for association with the diseases as calculated by the Armitage's trend test.

The two polymorphisms within IL8 were in very tight linkage disequilibrium in all populations (p = 0.00000 for all possible pairs). Thus we did not perform Bonferroni correction for multiple testing as it would be far too conservative in this case. In IL8RA M31R and R35C were transmitted together (p < 0.0001 for all populations).

### Haplotype analyses

The haplotype pattern in all populations is given in table 3. *IL8 *haplotypes showed weak association with asthma (p = 0.036). Furthermore, haplotypes of *IL8 *were significantly differently distributed between asthmatics and children with severe RSV infection (p = 0.005; see table 4). In contrast, no effect was seen with *IL8RA *haplotypes on either disease.

**Table 3:** Frequency of the haplotype within the three populations.

	**Haplotype**	**Asthma**	**RSV**	**Controls**
***IL8***	1 1	0.061	0.029	0.038
	1 2	0.380	0.443	0.455
	2 1	0.558	0.503	0.504
	2 2	0.002	0.024	0.004

***IL8RA***	1 1 1	0.904	0.922	0.897
	1 2 1	0.065	0.052	0.055
	2 1 2	0.031	0.026	0.044

Number 1 refers to the wildtype allele, 2 to the variant allele.

**Table 4:** Results of the haplotype analyses using FAMHAP.

	Asthma-Controls	RSV-Controls	RSV-Asthma
***IL8***	0.036	0.180	0.005
***IL8RA***	0.448	0.729	0.724

## Discussion

So far, only few studies have investigated the impact of polymorphisms within the IL8 signaling pathway on the genetic background of severe RSV associated diseases or bronchial asthma. The IL8 pathway includes *IL8 *itself, its two receptor chains *IL8RA *and *IL8RB *and its degradative enzyme aminopeptidase N [20]. In a previous study, we have shown that *IL8 *polymorphisms are in association with bronchial asthma in the German population using 230 asthmatic children and 270 randomly chosen controls [8]. Furthermore, we hypothesized that polymorphisms within *IL8 *have opposite effects on the development of asthma and severe RSV infections. The hypothesis was based on the observation that the -251T allele was more common in patients with asthma than in controls. In contrast, two studies from Hull and colleagues have demonstrated that the opposite allele, that is -251A, is associated with severe RSV infection in the English population [9,10].

Thus the first aim of the current study was to test whether our initial hypothesis of an opposite role of *IL8 *polymorphisms on asthma and RSV infections holds in a population of German children with severe RSV associated diseases. Second, we wanted to verify the association between bronchial asthma and *IL8 *polymorphisms in an extended asthmatic population (increased by 90 asthmatic children to 320 probands). Thirdly, we extended our investigation to include *IL8RA *as recently association of asthma and chronic obstructive pulmonary disease with this gene has been reported in another German samples [13].

In the here presented study, the polymorphisms -251A/T and -781C/T within *IL8 *showed no association with severe RSV associated diseases - neither in analyses of single polymorphisms nor in haplotype analyses. This might suggest that *IL8 *does not play a major role in the development of severe RSV infections in the German population. These findings are in contrast to the above mentioned studies of Hull *et al*. [9,10]. The discrepancy could be due to several reasons: The infants included in the study of Hull *et al*. were younger than in our study. Furthermore, the inclusion criteria differed slightly between both studies, for example, Hull and colleagues included children with pre-existing heart diseases, whereas we excluded those children. Finally, though the English and German population represent both Caucasians it is well known that the genetic predisposition to allergic diseases are at least partially different between Germans and English people [21].

The association between bronchial asthma and -781C/T within *IL8 *could be confirmed in an extended asthmatic population (p = 0.011), whereas the association with the second *IL8 *polymorphism -251A/T and asthma became weaker and was no longer statistically significant (p = 0.087). This might be in accordance to one study, showing that -251A/T has no functional impact, whereas the base pair substitution C to T at position -781 within the *IL8 *promotor enhances the binding of transcription factors and thus is probably more important in the genetic regulation of *IL8 *expression [22].

Furthermore, a direct comparison of the asthmatic population with the RSV population revealed significant differences, both for -781C/T alone (p = 0.034) as well as in the haplotype analyses (p = 0.005). These results may support our initial hypothesis, that polymorphisms within *IL8 *have opposite effects in the pathophysiology of asthma and RSV associated diseases. However, one should notice, that the allelic frequencies of the polymorphisms did not markedly differ between controls and children with severe RSV infections. Thus the positive association might merely reflect the fact that the genotype frequencies in asthmatics differ from controls.

In contrast to Stemmler *et al*, who found that the *IL8RA *polymorphisms M13R and R335C were significantly associated with bronchial asthma [13], we found no association of these polymorphisms neither with asthma nor severe RSV associated diseases. Again the conflicting results might be due to different inclusion criteria. The study by Stemmler *et al*. used 68 adult patients and 130 children with asthma whereas we used exclusively children with asthma. The "adult phenotype" of asthma is quite different from the asthmatic phenotype in children. For example pediatric asthma is much more often allergic than asthma in adults. Thus it might not be surprising that the genetic background of asthma differs between adults and children [23]. Furthermore, the size of our asthmatic study population was larger than the population of Stemmler *et al*. (198 asthmatic patients versus 320 patients). Thus the significant result in their study might just reflect a type 1 error. However, as the risk factor conferred by a single gene is quite small in complex genetic diseases like asthma, it is also possible that we missed true association in our population due to a type 2 error.

## Conclusion

We conclude from our data, that *IL8 *might play a role in the genetic predisposition to bronchial asthma and that these effects are different, or maybe even opposite to the effects of the same polymorphisms on severe RSV associated diseases. In contrast *IL8RA *polymorphisms do not play a major role, neither in the development of severe RSV infections nor in asthma.

## Competing interests

The author(s) declare that they have no competing interests.

## Authors' contributions

BP performed genotyping and sequencing of *IL8 *polymorphisms as well as the statistical analyses and drafted the manuscript.

MK participated in the clinical design of the study and the recruitment of the RSV population.

JH performed genotyping and sequencing of *IL8RA *polymorphisms

JF participated in the clinical design of the study.

AH conceived and coordinated the study and helped to draft the manuscript.

All authors have read and approved the final manuscript.
